# The Relationship between Health-Related Quality of Life, Subjective Scar Estimation, and Activity Performance in Adult Burn Patients 6 and 12 Months after Injury

**DOI:** 10.3390/ebj3040042

**Published:** 2022-10-05

**Authors:** Sara Enblom, Elin Sundin, Gerd Edvinsson Guné, Nona Aspling, Fredrik Huss

**Affiliations:** 1 Burn Center, Department of Plastic and Maxillofacial Surgery, Uppsala University Hospital, 751 85 Uppsala, Sweden; 2 Department of Surgical Sciences, Plastic Surgery, Uppsala University, 751 85 Uppsala, Sweden

**Keywords:** burn injury, occupational therapy, activity performance, health-related quality of life, scar evaluation

## Abstract

A burn injury affects a person’s health-related quality of life (HRQoL) in different ways and might influence their daily life for months and years afterward. The aim of this study was to examine how activity performance and subjective scar estimation relate to self-rated health and whether this changes in the first year post-burn. Fifty consecutive patients who were scheduled for follow-up at the Burn Center’s outpatient clinic in Uppsala were included. Assessments of HRQoL (EQ-5D), activity performance (DASH), and subjective scar evaluation (POSAS) were conducted at 6 and 12 months post-burn. The results show a statistically significant correlation between self-rated HRQoL and activity performance (*p* = 0.001) and between self-rated HRQoL and subjective scar estimation (*p* = 0.000) at 6 but not at 12 months post-burn. A possible explanation of the lack of correlation at one year post-burn might be the patient´s expectations of his or her recovery. In future research, it would be interesting to investigate the long-term correlations between quality of life and activity performance.

## 1. Introduction

A burn injury can affect physical function and daily activities in different ways [1]. Complications/sequlae such as pain, muscle weakness, scar contractures, and scar hypertrophy influence the quality of recovery considerably [2]. To be able to regain function and resume daily activities as soon as possible, rehabilitation should begin at an early stage. The need for mobilization, exercise, and scar treatment is often great and the need for rehabilitation normally remains for several years post-injury. Consequently, this may also affect the experience of health-related quality of life (HRQoL). When it comes to pain, Gauffin et al. [3] conclude that pain after burn injury often becomes chronic and that the HRQoL assessed early after a burn injury is a predictor for the development of chronic post-burn pain.

A burn injury also affects a person’s quality of life (QoL), even if the results vary between studies [4]. A review by Spronk et al. [5] demonstrated that most dimensions of HRQoL are affected shortly after the burn event and that the majority will recover over time, except for the dimensions physical and emotional role participation, anxiety, depression, and pain, which reflects the need for both mental and physical support after a burn injury. The result of a review by Giannoni-Pastor et al. shows a prevalence of psychological distress of up to 25% for more than two years after the burn injury [6]. One study mainly discussing small burns that were <5% of the total body surface area (TBSA) showed that the experienced QoL of patients at six months post-burn was close to equal to that of a normal population [7].

The terms HRQoL and QoL are often used interchangeably, but there is debate about their exact components and many different definitions can be found in the literature [8,9]. Both have been defined as physical, social, material, and emotional well-being. One definition of QoL also includes economic and political values, whereas HRQoL focuses more on health and does not include non-health-related aspects [9]. Post et al. [8] found four broad health dimensions that are often incorporated in QoL definitions: physical health, mental health, social health, and functional health. The Euro QoL five-dimensions questionnaire (EQ-5D) measures the health-related quality of life (HRQoL), with more emphasis on health issues.

Regardless of the definition, it is valuable to clarify and compare the individual experience of quality of life [8]. QoL and HRQoL are always assessed using a self-assessment scale. However, a guideline on the measurement of HRQoL, specifically in burn patients, is currently missing [5]. The three most applied instruments, measuring HRQoL and also validated in the burn population, are the burn-specific health scale brief (BSHS-B), the medical outcome study short form, with 36 items (SF-36), and the Euro QoL five-dimensions questionnaire (EQ-5D) [5]. Furthermore, the Boston outcomes questionnaire [10] and the Brisbane burn-scar impact scale [11] are other examples of assessment tools used to determine the long-term impact of post-burn scarring on life quality [11,12].

Aghajanzade et al. show in their study that patients with hand burns experience a great impact on hand functionality early post-burn [13]. Research about the patients’ subjective experience of hypertrophic scars regarding hand function and/or activity performance has not been found in the literature. However, research about the influence of hypertrophic scars on quality of life gives some more results. A significant negative correlation was observed between QoL and the results of the patient and observer assessment scale (POSAS) in a study of 41 patients who received allotransplants, which implies that greater dissatisfaction with scarring leads to a lower QoL [14]. A significant difference was reported in a study of people with burn scars versus non-burn scars, where the burn-scar group reported that the scars made them feel isolated and gave them unwanted attention to a significantly higher degree compared to the non-burn scars group [15]. Contrary results are reported in a Norwegian study, where, almost four years after healing, the patients still showed significant impairment of generic health compared to a general population but no deterioration in overall quality of life [16].

There is a lack of knowledge regarding the possible correlations between hypertrophic scarring and activity performance and the results regarding the impact on QoL are variable. Therefore, the aim of this study was to examine how activity performance and subjective scar estimation in burn patients relate to self-rated HRQoL and if this changes over time.

## 2. Materials and Methods

During a 15-month period, all patients who came for follow-up appointments at the Burn Center at Uppsala University Hospital at 6 and 12 months post-burn, and who fulfilled the inclusion but no exclusion criteria, were asked to participate by any of the treating occupational or physical therapists. The patients received oral and written information about the study and were always informed about the possibility, at any time point, of ending their participation without needing to give a reason. The inclusion criteria were adults ≥ had been in-patients at the Burn Center (minimum 24 h) and who had mastered Swedish verbally and in writing. Exclusion criteria were if any underlying disease (for example, dementia, mental illness, stroke, or conditions that affect function) makes it impossible to assess the impact of the burn on the function and/or QoL. During the follow-up visits, several assessments and tests were carried out by the treating occupational therapist, using the EQ-5D, the disabilities of the arm, shoulder, and hand questionnaire (DASH), and the patient and observer scar assessment scale (POSAS).

EQ-5D is a standardized assessment tool for measuring self-assessed health-related quality of life and the current state of health [17]. The assessment is divided into two parts; part one is a self-estimate in five dimensions: mobility, self-care, usual activities, pain and discomfort, anxiety, and depression. Every dimension has three answer options. Part two, EQ VAS, is an estimation of the total current state of health using a visual analog scale (VAS) where 0 is “the worst health you can imagine” and 100 is “the best health you can imagine”. EQ-5D shows high clinical validity and good psychometric values in Swedish burn patients [18]. EQ VAS was used in this study [17].

Disabilities of the Arm, Shoulder, and Hand Questionnaire (DASH) is a self-assessment scale. Part one of the questionnaire consists of 30 questions where the patient rates the ability to perform hand- and arm-related daily activities and how this affects daily life. Part two is optional and covers work and leisure activities [19]. The assessment is based on the World Health Organization´s (WHO) ”International Classification of Functioning, Disability, and Health” (ICF) and explores impairments, activity limitations, and participation restrictions [20,21]. The assessment is valid and reliable. The Swedish version shows high internal consistency, excellent test-retest reliability, and good content validity [22]. However, the assessment has not, to our knowledge, been used scientifically in the burn population previously. This assessment was only issued to the 37 patients who had a burn injury to the arm, shoulder, or hand.

The Patient and Observer Scar Assessment Scale (POSAS) consists of two scales, the observer scale and the patient scale. In this study, only the patient scale is included. The patient scale consists of six items (pain, itch, color, stiffness, thickness, and irregularity). These six items are summed up to a total score. The patient is also asked to give an overall opinion, which is scored from 1 to 10. The POSAS is valid and reliable for burn scars [23] and has recently been translated to Swedish and approved by the POSAS group.

The results of these assessments were used in the study. Descriptive data and correlations were analyzed with Pearson´s correlation and Spearman’s *rho*, using IBM SPSS Statistics for Windows (version 23, IBM Corp, Armonk, NY, USA). Records of the participants’ age, gender, and TBSA percentage (TBSA%) were collected from medical records. All the included individuals have given their informed consent and the study has been approved by the Uppsala regional ethical review board (Dnr 2015/411).

## 3. Results

During the study period, 72 consecutive patients came for follow-ups at the Burn Center. Twenty-two did not meet the inclusion criteria: fourteen due to underlying diseases, two declined, two did not fulfill the language criteria, two did not fulfill the in-patient criteria, one was excluded due to their general condition, and one failed to respond. This left a total of 50 patients, 35 men (70%) and 15 women, who agreed to participate.

The included patients were aged 18 to 80 years (mean = 44.5, SD = 16.5), with a burn injury of 0.25 to 83.5 TBSA% (mean = 13.5, median = 7.5, SD = 14.7). The etiology of the burns was 72% from flames, 18% from scalds, 6% were chemical, and 4% from an electrical injury. Eight patients (16%) had full-thickness burns only, 16 patients (33%) had partial-thickness burns only, and 25 patients (51%) had both full- and partial-thickness burns. The data set (TBSA%) taken from one patient was missing. In total, 37 patients (74%) had burns to their upper limbs. Data from 30 of these respondents were gathered at the 6-month follow-up appointment, with data from 27 patients gathered at 12 months. The length of stay during their initial in-patient care at the burn center varied from 1 to 113 days (mean = 17.4, median = 14, SD = 19.9). The results of the assessments are displayed in Table 1.

There was a statistically significant correlation at 6 months post-burn between self-rated health (EQ VAS) and activity performance (DASH) (*p* = 0.001) and with each patient’s subjective scar assessment (POSAS) (*p* < 0.001). The statistically significant correlations at 6 months were, however, not found at 12 months. There was no statistically significant correlation between self-rated health (EQ VAS) and TBSA% on either of the occasions (Table 2).

Comparisons over time showed that 12/20 patients (60%) gave better ratings (lower value) on their activity performance (DASH) at 12 months post-burn, compared to 6 months (Table 3). Seven patients (35%) felt that their activity performance was worse, and 1 patient (5%) gave an equal estimate. When it came to subjective scar assessment (POSAS), 10 out of 25 patients (40%) believed that their scars had improved at 12 months compared to 6 months. Five patients (20%) experienced a deterioration, and 10 patients (40%) thought that the scars were unchanged. Thirty-two patients fulfilled the QoL health assessment (EQ VAS) at both visits. Fourteen of them (44%) believed that their quality of life had improved at 12 months compared to 6 months. Nine patients (28%) rated their QoL lower, and 9 patients (28%) gave an equal estimate (Table 3).

The number of participants was low in some of the subgroups, which makes the results uncertain and not possible to generalize. However, 35% (7/20) rated their activity performance, according to DASH, to be less/inferior at 12 months compared to 6 months. Due to the small subgroups, no statistical analyses were performed. However, a trend toward a more reduced activity performance at 12 months is notable.

## 4. Discussion

The aim of this study was to examine how activity performance and subjective scar estimation relate to self-rated health. The assessments are the ones that are routinely used in the outpatient clinic at the Burn Center in Uppsala. They were originally chosen for being reliable in the burn population, all except for DASH. An assessment tool of activity performance that was specifically designed for Swedish burn patients was not found in the literature; since the DASH assessment has shown good validity and excellent reliability in other patient groups in Sweden, it was selected.

Thirty-six patients were included at the 6-month follow-up and 14 at the 12-month follow-up appointments. Data from both visits are included for all patients, as far as possible. This explains why some of the results of EQ VAS and POSAS are missing since the data set was not complete when gathered retrospectively from the medical records. Seven patients did not appear at their 12-month follow-up, for different reasons. This is also why the number of patients differs between follow-ups. The difference in numbers regarding DASH is due to the fact that not all patients had a burn injury to the arm, shoulder, or hand and, consequently, did not fill out the DASH questionnaire. The reason for using DASH in this study at all originates from an earlier routine at the burn center. After this study was completed, this routine has been supplemented with a more overall activity performance measure, “Performance and Satisfaction in Activities of Daily Living” (PS-ADL) [24].

The results show an expected correlation at 6 months post-burn between self-rated health, activity performance, and the patients´ subjective scar assessment. Less expected was the absence of correlation at 12 months post-burn. This is contrary to the results of Nitescu et al. [14], who found a strong relationship between the WHOQOL-BREF (World Health Organization quality-of-life instrument) questionnaire and the POSAS scale at 12 months post-burn.

Our results might cohere with the comparisons over time. Sixty percent of the patients reported that their activity performance had increased at 12 months, but a surprisingly high percentage, 40%, felt that it has decreased, or is equal, compared to the result at 6 months. Even more surprising is the result of EQ VAS, where 56% rate their quality of life as equal or worse at 12 months. Possible explanations for this might be that the patient’s expectations affect the result. At 6 months, most patients are grateful to be able to cope, to have left the hospital, and to manage the most necessary everyday tasks. At 12 months, many expect life to be back to normal and they may feel disappointment and letdown when it is not. Back home, they suddenly discover capacities that are lacking and face the realization that certain functions might never be restored. Hobbies and sports demand too much from them and a return to work seems remote, but, as Quinn et al. conclude in their review, the factors influencing the return to work are many, complex, and interlinked [25].

When it comes to scarring, 60% estimate their scars to be equal or worse at 12 months. This might depend on the scarring process, which, for most people, is longer than one year, and is sometimes two years or more [26]. Druery et al. found that scores in the physical domain of BSHS-B (the burn-specific health scale brief), even two years post-burn, significantly increased when the TBSA was more than 60% [27]. Thus, it is not so surprising that the patients in this study, after only one year, perceive that the scars are not satisfactory, despite the fact that the patients in this study had comparatively smaller burns. Therefore, in future research, it would be interesting to investigate more long-term correlations.

The lack of statistically significant correlations between self-rated health (EQ VAS) and TBSA% on both occasions may seem unexpected. One possible explanation for this is that patients have different ways of coping with their injury and its complications, depending on the individual’s conditions and coping strategies, different needs, and demands. This means that a person with a relatively small burn injury might experience a greater impact on their QoL than someone with a larger injury. The location of the scar, or if, for example, it causes contractures, can affect the patient’s QoL more than the actual size of the scar [28]. The location of the patients’ scars was not registered in this study. It would have been interesting to see if the burn site also correlates to QoL among our patients. Nor were any statistical correlations made between the etiology of the burns and QoL, since the subgroups would have been very small. A review by Spronk et al. [5] did not find any associations between etiology and HRQoL. In a long-term study by Moi et al., the authors conclude that the overall quality of life (measured by the quality of life scale) improved significantly as much as 11 years later [29], which confirms that the rehabilitation period is long. The usual routine at our burn center, when patients express low values of QoL or mention other needs that cannot be met by the team, is to refer them either to the social worker, the dietician, or the psychologist linked to our burn center, or to a corresponding support worker closer to the patient’s place of residence.

The result of this study gives more information on the patients’ experiences following a burn injury. It gives an understanding of the development after a burn injury; this knowledge will benefit patients in the future and improve/strengthen the information given to them, help them to develop new routines, and give them more realistic expectations of their recovery.

The major limitation of this study is the relatively small sample size; the fact that the subgroups are small, 1–14 people, makes the results unreliable. No statistical analyses have, therefore, been performed in these groups. The use of DASH in evaluating patients’ daily activities further reduces the number of patients since not all included patients sustained a burn to the upper limb. A larger sample/cohort and a multi-center collaboration would be desirable for a more generalizable result in future research. Another difficulty is the assessment scale (POSAS) and the fact that the instrument was new to the team at the time of the follow-ups; therefore, diverging instructions might have been given to the patients. The study design, with both retrospective and prospective data collection, proved to be a challenge since not all the desirable data were available retrospectively from the 6-month follow-up point with those patients who were included at 12 months.

## 5. Conclusions

The results of this study indicated that the ability to carry out personal daily activities affected burn patients´ perceived health early post-burn. The same goes for the patients´ subjective scar evaluations. The small sample size must be taken into consideration when interpreting these results.

## Figures and Tables

**Table 1:** Assessments at 6 and 12 months post-burn trauma.

Assessment (Range)	6 Months Post-Burn		12 Months Post-Burn	
	*n*	Mean (Range) Median (SD)	*n*	Mean (Range) Median (SD)
**DASH (0–100)**	30	19.6 (0–68.8) 10.8 (19.9)	27	15.4 (0–54.2) 5.8 (17.8)
**EQ VAS (0–100)**	44	74.3 (20–100) 80.0 (19.2)	38	75.6 (30–100) 80.0 (15.8)
**POSAS Patient, overall (1–10)**	38	6.6 (1–10) 6.0 (2.6)	37	6.6 (2–10) 7.0 (2.2)

**Table 2:** Correlations with the self-rated health (EQ VAS) and otherassessments.

	6 Months Post-Burn			12 Months Post-Burn		
	*n*	*p*	r	*n*	*p*	r
**DASH ^1^**	28	0.001 **	−0.613	27	0.135	−0.295
**POSAS ^1^**	38	0.000 **	−0.552	37	0.087	−2.85
**TBSA% ^2^**	43	0.841	0.032	37	0.270	−0.186

^1^ Spearman´s rho. ^2^ Pearson´s correlation; ** Correlation is significant at the 0.01 level (2-tailed).

**Table 3:** Comparisons over time.

	Total (*n*)	Negative Ranks (*n*)	Negative Ranks (Mean)	Positive Ranks (*n*)	Positive Ranks (Mean)	Ties
**DASH**	20	12	11.4	7	7.57	1
**POSAS**	25	10	8.90	5	6.20	10
**EQVAS**	32	9	10.8	14	12.8	9

Negative ranks: 12 months < 6 months. Positive ranks: 12 months > 6 months. Ties: equal rating.

## Data Availability

The data presented in this study are available on request from the corresponding author.
